# Crosstalk between regulatory non-coding RNAs and oxidative stress in Parkinson’s disease

**DOI:** 10.3389/fnagi.2022.975248

**Published:** 2022-08-09

**Authors:** Hantao Zhang, Xiaoyan Liu, Yi Liu, Junlin Liu, Xun Gong, Gang Li, Min Tang

**Affiliations:** ^1^School of Life Sciences, Jiangsu University, Zhenjiang, China; ^2^Institute of Animal Husbandry, Jiangsu Academy of Agricultural Sciences, Nanjing, China; ^3^Department of Rheumatology & Immunology, Affiliated Hospital of Jiangsu University, Zhenjiang, China; ^4^Department of Vascular Surgery, The Second Affiliated Hospital of Shandong First Medical University, Taian, China

**Keywords:** Parkinson’s disease, microRNAs, long non-coding RNAs, circular RNAs, oxidative stress

## Abstract

Parkinson’s disease is the second most common neurodegenerative disease after Alzheimer’s disease, which imposes an ever-increasing burden on society. Many studies have indicated that oxidative stress may play an important role in Parkinson’s disease through multiple processes related to dysfunction or loss of neurons. Besides, several subtypes of non-coding RNAs are found to be involved in this neurodegenerative disorder. However, the interplay between oxidative stress and regulatory non-coding RNAs in Parkinson’s disease remains to be clarified. In this article, we comprehensively survey and overview the role of regulatory ncRNAs in combination with oxidative stress in Parkinson’s disease. The interaction between them is also summarized. We aim to provide readers with a relatively novel insight into the pathogenesis of Parkinson’s disease, which would contribute to the development of pre-clinical diagnosis and treatment.

## Introduction

Parkinson’s disease (PD) is a common neurologic disease, which affected about 6.1 million people around the world in 2016 (Bloem et al., 2021). The elderly are more likely to suffer from this disease, with most cases occurring after the age of 50 (Opara et al., 2017). PD is mainly characterized by the loss of dopaminergic neurons and the presence of Lewy bodies in surviving neurons, while its exact cause is unknown (Antony et al., 2013; Raza et al., 2019; Li W. et al., 2020). The disease can be intolerable because it is a progressive disease and can severely damage the somatic motor system, making it difficult for PD patients to use their hands or walk normally (Schneider et al., 2017; Cerri et al., 2019; Hayes, 2019). They may also exhibit multiple non-motor symptoms, including cognitive decline, depression, anxiety, and sleep disorders (Reich and Savitt, 2019). A number of studies illustrated that many types of RNA have been deeply involved in the whole disease progression even after onset, including but not limited to long non-coding RNAs (lncRNAs), microRNAs (miRNAs), and circularRNAs (circRNAs; Choi et al., 2018, Liu N. et al., 2020; Liu et al., 2021; Wu and Kuo, 2020; Wang W. et al., 2021). All of the three most well-known and most commonly studied RNAs belong to the regulatory non-coding RNAs (ncRNAs), a category of ncRNAs that are transcribed from DNA and not involved in coding proteins (Szymański and Barciszewski, 2002).

lncRNAs are a collective term for a group of highly heterogeneous regulatory ncRNAs (Hombach and Kretz, 2016). The common characteristics of lncRNA mainly include transcripts longer than 200 and a lack of ability to encode proteins (Yang et al., 2021). Given their wide variety, their biological roles can be quite diverse. An important function of lncRNA is to regulate gene expression at the transcriptional, post-transcriptional, and epigenetic levels (Panni et al., 2020). lncRNAs can bind certain RNAs and proteins to prevent them from interacting with other molecules (Wang, 2018). They can also affect epigenetic modifications of genes and histones. In addition, several lncRNAs have been found to have a role in the maintenance of chromosome stability and the regulation of the cell cycle (Lee et al., 2016; Munschauer et al., 2018; Wang R. et al., 2018; Zhang et al., 2018).

miRNAs, small RNA molecules with an average length of 22 nt, must be the most extensively studied and best understood small non-coding RNAs (sncRNAs; Zhang P. et al., 2019). The major function of miRNAs is to control mRNA translation, which is usually based on the complementary base pairing between seed regions of miRNAs and 3’ untranslated regions of mRNAs (Wei J. W. et al., 2017). The miRNA first incorporates Argonaute proteins to form an RNA-induced silencing complex (RISC; Scott and Ono, 2011). Then, if miRNAs show imperfect complementarity to their target mRNAs, which is usually the case in animals, deadenylation of the mRNA will occur, leading to translation inhibition (Carvalho Barbosa et al., 2020). Furthermore, a small number of miRNAs can upregulate gene expression, though this process is fairly rare (Gerin et al., 2010).

circRNAs, a unique group of regulatory ncRNAs, are characterized by their covalently closed structures (Zhou W. Y. et al., 2020). Due to a lack of capping and polyadenylation, circRNAs are resistant to RNA exonucleases and more stable than linear RNAs (Huang et al., 2020). The size of circRNA range from 100 nt to over 4 kb, so it might belong to lncRNA and sncRNA at the same time (Zhang P. et al., 2019). Increasing evidence has demonstrated that a subset of circRNAs exerts their functions by functioning as competing endogenous RNA (ceRNA) or miRNA sponges (Cao et al., 2020; Jiang Q. et al., 2020; Li L. et al., 2020; Peng et al., 2021). miRNAs captured by circRNAsare unable to regulate gene expression (Jin et al., 2020). In addition, several circRNAs can act as protein decoys. Through the interaction with these circRNAs, the biological functions of a variety of proteins can be changed (Altesha et al., 2019).

In addition to the aforementioned regulatory ncRNAs, oxidative stress is believed to have a notable effect on the pathological progression of PD (Wang X. et al., 2020). It results from an imbalance between oxidant production and antioxidative defenses (Forman and Zhang, 2021). The oxidant refers to reactive oxygen species (ROS) and reactive nitrogen species (RNS), which are mainly produced in mitochondria (Turrens, 2003). ROS includes superoxide (O^•–^_2_), hydrogen peroxide (H_2_O_2_), hydroxyl radical (•OH), ozone, and singlet oxygen. These small molecules are all derived from the reaction of oxygen with electrons (Brieger et al., 2012). The primary intermediate in the biosynthesis of RNS is nitric oxide (NO; Lushchak and Lushchak, 2021). NO reacts with ROS, thereby giving rise to other forms of RNS such as peroxynitrite and peroxynitrous acid (ONOOH; Tharmalingam et al., 2017). Disruption of the ATP production function of mitochondria as well as inflammation may cause an increase in oxidant production (Brand and Nicholls, 2011; Islam, 2017). In response to ROS and RON, organisms have evolved antioxidant defense systems. Such antioxidant defenses are largely provided by antioxidant enzymes, including superoxide dismutase (SOD), catalase (CAT), and glutathione peroxidase (GPx; Prasad et al., 2017). By catalyzing specific reactions, these enzymes can scavenge oxidants and repair oxidative damage. Excessive oxidant production combined with decreased expression and activity of antioxidant enzymes will induce oxidative stress, which can greatly impair cell viability (Nunomura et al., 2012; Chen and Zhong, 2014).

It is undeniable that a lot of research has been conducted on the role of regulatory ncRNAs or oxidative stress in PD (Lu et al., 2017, 2019; Pavlou and Outeiro, 2017; Cao et al., 2019; Nies et al., 2021; Zhu et al., 2021). However, the interaction between the minPD has not been well studied. It is worth noting that both regulatory ncRNAs and oxidative stress are closely involved in neurodegenerative disorders (Nunomura and Perry, 2020). On the one hand, oxidative stress can cause damage to nucleic acids and affect the expression levels of varieties of regulatory ncRNAs (Radi et al., 2014). On the other hand, several regulatory ncRNAs have the potential to regulate oxidative stress-related pathways, which represent promising therapeutic targets (Geng et al., 2017; Song et al., 2019; Li Y. et al., 2020; Li et al., 2021). Hence, further research on this molecular mechanism in PD may be beneficial to our understanding of the neurodegenerative disorder in conjunction with the development of novel strategies for pre-clinical diagnosis and therapeutic intervention.

## How Do The Ncrnas Regulate Parkinson’s Disease?

Once it was thought that ncRNAs did not have any biological function. Only recently has it been discovered that the roles of RNAs are not limited to bridging genes and proteins (Carvalho Barbosa et al., 2020). The last decade has witnessed the discovery and annotation of thousands of both housekeeping and regulatory ncRNAs, which are emerging as key regulators of gene expression at the transcriptional or post-transcriptional level (Li et al., 2015; Merry et al., 2015; Yildirim et al., 2020; Rezaei et al., 2021). Several subtypes of regulatory ncRNAs, including miRNAs, lncRNAs, and circRNAs, are involved in the pathogenesis of PD (Majidinia et al., 2016). The PD models used and the regulatory ncRNAs identified are shown in Table 1.

**Table 1:** Summary of regulatory non-coding RNAs that regulate Parkinson’s disease.

Model/ Cell Type	Name	Target	Potential Role	References
Human pluripotent H9 cells and neural progenitor ReNcell VM cells, HEK293T cells, C57BL/6J mice	miRNA-7 ↓	SNCA	Suppressed α-synuclein expression	(McMillan et al., 2017; Choi et al., 2018; Adusumilli et al., 2020)
C57BL/6 mice	miRNA-26a ↓	DAPK1	Alleviated DA neuron loss	(Su et al., 2019)
A mouse model of PD	miRNA-29b2/c ↓	AMPK	Promoted neuroinflammation	(Bai et al., 2021)
SH-SY5Y cells, C57BL/6 mice	miRNA-29c ↓	SP1, NFAT5	Attenuated the neuroinflammation and apoptosis of PD	(Wang X. et al., 2020)
C57BL/6 mice	miRNA-30a-5p ↑	PKCα	Downregulated GLT-1 and caused glutamate excitotoxicity	(Meng X. et al., 2021)
Human neuroblastoma SH-SY5Y cells	miRNA-30b ↓	SNCA	Inhibited MPP+-induced neuronal apoptosis	(Shen et al., 2020)
C57BL/6 mice	miRNA-30e ↓	Nlrp3	Attenuated neuroinflammation	(Li D. et al., 2018)
BV-2 and HEK-293T cells, C57BL/6J mice	miRNA-93 ↓	STAT3	Reduced neuronal injuries and suppressed inflammatory reaction	(Wang X. et al., 2021)
C57BL/6J mice	miRNA-103a-3p ↑	Parkin	Prevented mitophagy	(Zhou J. et al., 2020)
C57BL/6 mice	miRNA-124 ↓	MEKK3, EDN2	Inhibited neuroinflammation and suppressed neuronal apoptosis	(Yao et al., 2018; Wang J. et al., 2019)
C57BL/6 mice	miRNA-128 ↓	AXIN1	Reduced DA neuron apoptosis	(Zhou et al., 2018)
Human SH-SY5Y cells, a mouse model of PD	miRNA-132 ↑	SIRT1	Induced apoptosis	(Qazi et al., 2021)
PD patients, C57BL/6J mice, BV-2 microglial cells	miRNA-132-3p ↑	GLRX	Aggravated neuroinflammation	(Gong et al., 2022)
C57BL/6 mice	miRNA-132-5p ↑	ULK1	Induced autophagy	(Zhao et al., 2020a)
PC-12 rat adrenal pheochromocytoma cells	miRNA-133a ↓	RAC1	Inhibited cell apoptosis and autophagy	(Lu et al., 2020)
PC-12 cells	miRNA-133b ↓	ERK1/2	Inhibited nerve cell apoptosis	(Dong et al., 2020)
Human neuroblastoma SK-N-SH cells	miRNA-181a ↓	p38, JNK	Inhibited apoptosis and autophagy	(Liu Y. et al., 2017)
PC-12 cells	miRNA-181b ↓	PTEN	Inhibited autophagy and promoted cell viability	(Li W. et al., 2018)
PC-12 cells	miRNA-181c ↓	BCL2L11	Inhibited cell apoptosis and promoted cell viability	(Wei M. et al., 2017)
C57BL/6 mice	miRNA-183 ↑	OSMR	Promoted the apoptosis of substantia nigra neurons	(Gao et al., 2019)
Human neuroblastoma SH-SY5Y cells	miRNA-185 ↓	AMPK, mTOR	Inhibited autophagy and apoptosis of dopaminergic cells	(Wen et al., 2018)
BV2, HEK293, and SH-SY5Y cells, C57BL/6J mice	miRNA-190 ↓	Nlrp3	Alleviated neuronal damage and inhibited inflammation	(Sun et al., 2019)
BV2 microglial cells	miRNA-195 ↓	ROCK1	Inhibited neuroinflammation	(Ren et al., 2019)
MPP+-induced mouse model of PD	miRNA-199a ↓	GSK3β	Reduced autophagy and alleviated PD-related phenotypes	(Ba et al., 2020)
Humandopaminergic neuroblastoma SH-SY5Y cells	miRNA-216a ↑	Bax	Reduced MPP+-induced neuronal apoptosis	(Yang et al., 2020)
Ratadrenal pheochromocytoma PC-12cells	miRNA-221 ↓	PTEN	Promoted cell proliferation and inhibited cell apoptosis	(Li L. et al., 2018)
C57BL/6 mice	miRNA-330 ↑	SHIP1	Suppressed chronic neuroinflammation	(Feng et al., 2021)
SH-SY5Y cells, C57BL/6 mice	miRNA-384-5p ↑	SIRT1	Promoted the progression of PD	(Tao et al., 2020)
Mouse embryonic substantial nigra–derived SN4741 cells	miRNA-421 ↑	MEF2D	Promoted DA neuron death	(Dong et al., 2021)
HEK293T, SH-SY5Y, and U87 cells	miRNA-486-3p ↓	SIRT2	Reduced α-Syn aggregation and suppressed α-Syn	(Wang Y. et al., 2018)
C57BL/6 mice	miRNA-543-3p ↑	Slc1a2	Down-regulated GLT-1 and caused glutamate excitotoxicity	(Wu X. et al., 2019)
C57BL/6 mice	miRNA-599 ↓	LRRK2	Suppressed cell apoptosis	(Wu Q. et al., 2019)
A mouse model of PD	miRNA-873 ↑	A20	Aggravated neuroinflammation	(Wu et al., 2020)
C57BL/6 mice	miRNA-7116-5p ↓	TNF-α	Prevented loss of DA neurons	(He et al., 2017)
BV2 microglial cells, C57BL/ 6 mice	miRNA-let-7a ↓	STAT3	Inhibited microglial activation and inflammation	(Zhang J. et al., 2019)
MN9D cells	miRNA-let-7d ↓	Caspase-3	Enhanced cell viability and inhibited cell apoptosis	(Li et al., 2017)
Human dopaminergic neuronal SH-SY5Y cells, a mouse model of PD	BDNF-AS ↑	miRNA-125b-5p	Inhibited cell apoptosis and autophagy	(Fan Y. et al., 2020)
BV2 microglia cells, C57BL/ 6 mice	GAS5 ↑	miRNA-223-3p, miRNA-150	Promoted the release of inflammatory cytokines and contributed to the neuron loss	(Xu et al., 2020; Ma et al., 2022)
N27 dopaminergic neurons, C57BL/6 mice	H19 ↓	miRNA-301b-3p, miRNA-585-3p	Protected against dopaminergic neuron loss	(Jiang J. et al., 2020)
Human neuroblastoma SH-SY5Y cells, C57BL/6 mice	HOTAIR ↑	miRNA-126-5p	Induced cell apoptosis	(Wang et al., 2017; Lin et al., 2019)
Dopaminergic neuron SH-SY5Ycells and BV2 microglial cells, C57BL/6 mice	HOXA11-AS ↑	miRNA-124-3p	Induced neuroinflammation	(Cao et al., 2021)
Human neuroblastoma SK-N-SH cells, C57BL/6 mice	LINC-00943 ↑	miRNA-7-5p	Regulated the apoptosis and inflammation of nerve cells	(Meng C. et al., 2021; Sun et al., 2022)
Human neuroblastoma SH-SY5Y cells, C57BL/6 mice	LincRNA-p21 ↑	miRNA-1277-5p	Inhibited viability and promoted apoptosis of cells	(Xu et al., 2018)
Human neuroblastoma SK-N-SH, SK-N-BE, and SH-SY5Y cells, human embryonic kidneyHEK293 cells, MN9D dopaminergic neuronal cells, C57BL/6 mice	MALAT1 ↑	miRNA-135b-5p, miRNA-124, miRNA-205-5p	Promoted cell apoptosis	(Liu W. et al., 2017; Chen Q. et al., 2018; Lv et al., 2021)
BALB/c mice	MIAT ↓	miRNA-34-5p	Exerted neuroprotective effects in PD	(Shen et al., 2021a)
Human neuroblastoma SH-SY5Y, SK-N-SH, and SK-N-AS cells, embryonic kidney epithelialHEK293T cells, C57BL/6 mice	NEAT1 ↑	miRNA-124, miRNA-212-5p, miRNA-1301-3p, miRNA-519a-3p, miRNA-213-3p	Promoted inflammatory response and neuronal apoptosis	(Yan et al., 2018; Xie S. P. et al., 2019; Liu R. et al., 2020, Liu et al., 2021; Chen M. Y. et al., 2021)
SH-SY5Y cells	OIP5-AS1 ↓	miRNA-137	Promoted mitochondrial autophagy, reduced the level and toxicity of α-syn	(Song and Xie, 2021; Zhao et al., 2022)
Human neuroblastoma SK-N-SH, SK-N-AS cells, SH-SY5Y cells, MN9Ddopaminergic neurons, C57BL/6 mice	SNHG1 ↑	miRNA-7, miRNA-15b-5p, miRNA-181a-5p, miRNA-221/222, miRNA-216-3p	Affected neuroinflammation, autophagy, and apoptosis in PD	(Cao et al., 2018; Chen Y. et al., 2018; Qian et al., 2019; Wang C. et al., 2021; Wang et al., 2021a, b)
C57BL/6 mice	SNHG14 ↑	miRNA-214-3p	Exacerbated damage to DA neurons, accelerated the progression of PD	(Zhang L. M. et al., 2019; Zhou S. et al., 2020)
SH-SY5Ycells, C57BL/6 mice	UCA1 ↑	miRNA-423-5p	Promoted α-Syn accumulation	(Lu et al., 2018)
Human neuroblastoma SH-SY5Y cells and rat adrenal pheochromocytoma PC-12 cells	XIST ↑	miRNA-199a-3p	Contributed to the apoptosis of DA neurons	(Zhou Q. et al., 2021)
Human neuroblastomaSH-SY5Y cellsand mice dopaminergic neuronal MN9D cells, C57BL/6 mice	circDLGAP4 ↓	miRNA-134-5p	Induced apoptosis and enhanced autophagy	(Feng et al., 2020)
SH-SY5Yneuroblastoma cells and BV-2 microglial cells, C57BL/6 mice	circSAMD4A ↑	miRNA-29c-3p	Affected the apoptosis and autophagy of DAs	(Wang W. et al., 2021)

The upward arrows indicate that the levels of non-coding RNAs are significantly higher in PD patients than in healthy individuals and vice versa.

### Effects of miRNAs in Parkinson’s disease

PD is characterized by the loss of dopaminergic neurons (DAs) in the substantia nigra (Goh et al., 2019). Convincing evidence indicated that neuroinflammation was involved in DA death (Gordon et al., 2018; Rodriguez-Perez et al., 2018). Activated microglia may initiate the inflammatory process in the central nervous system (CNS). The upregulation of microRNA-132-3p (miR-132-3p) and microRNA-873 (miR-873) in PD led to the deficiency of ATP-binding cassette transporter A1 (ABCA1) and GLRX, which may give rise to the activation of microglial cells and subsequent neuronal death (Wu et al., 2020; Gong et al., 2022). microRNA-29b2/c (miR-29b2/c) and microRNA-124 (miR-124) played contrasting roles in microglia activation. Knockout of miR-29b2/c would inhibit microglia activation, whereas decreased expression of miR-124 correlated with the progression of microglia activation (Yao et al., 2018; Bai et al., 2021). Feng et al. found that activated microglia can be transformed into two different phenotypes, i.e., M1 polarization and M2 polarization. M1 microglia could produce proinflammatory cytokines to maintain the homeostasis of the central nervous system. However, this process may be prolonged by microRNA-330 (miR-330), which in turn caused inflammatory damage to neuronal cells (Feng et al., 2021). TNF-α was a proinflammatory factor released by M1 polarization, whose production was downregulated by micro-RNA-7116-5p (miR-7116-5p; He et al., 2017). Nod-like receptor protein 3 (Nlrp3) functioned in regulating proinflammatory cytokines. Both microRNA-30e (miR-30e) and microRNA-190 (miR-190) targeted the 3’ UTR of Nlrp3 mRNA and thus inhibited neuroinflammation (Li et al., 2018; Sun et al., 2019). Furthermore, Specific protein-1 (SP1), nuclear factor of activated T cells 5 (NFAT5), Rho-associated kinase 1 (ROCK1), and transcriptional activator 3 (STAT3) participated in regulating neuroinflammation. By suppressing the expression of SP1, NFAT5, ROCK1, and STAT3, microRNA-29c (miR-29c), microRNA-195 (miR-195), microRNA-93 (miR-93), and microRNA-let-7a (miR-let-7a) may reduce neuronal damage caused by inflammatory responses (Ren et al., 2019; Zhang et al., 2019; Wang et al., 2020a, b; Wang et al., 2021).

Another hallmark of PD is the formation of Lewy bodies in neurons (Singh and Sen, 2017). The main component of Lewy bodies was α-synuclein fibrils (α-syn) toxic to DA neurons. Apoptosis was induced when the formation of α-syn aggregates exceeded the limit of what the cell can tolerate, suggesting α-syn is an essential player in the PD neurodegenerative process (Rocha et al., 2018). miRNA-7 (miR-7) was found to not only inhibit α-syn expression by interacting with its messenger RNA (mRNA) but also help remove α-syn by promoting autophagy (Choi et al., 2018). As McMillan et al. stated in their study, miR-7 may promote autophagy by suppressing the expression of epidermal growth factor receptor (EGFR), since the activated EGFR was able to inhibit autophagy. Then, α-syn can be degraded by autophagy (McMillan et al., 2017). In addition to promoting α-syn degradation, researchers found that two RNAs affected the toxicity of α-syn. Both phosphorylation and acetylation were essential mechanisms regulating the neurotoxicity of α-syn. Su et al. discovered that miRNA-26a (miR-26a) directly downregulated the expression level of death-associated protein kinase 1 (DAPK1), and DAPK1 was capable of promoting the phosphorylation of α-syn (Su et al., 2019). Also, sirtuin 2 (SIRT2) was identified to catalyze the deacetylation reaction of α-syn, and miRNA-486-3p (miR-486-3p) may affect the toxicity of α-syn by regulating the expression of SIRT2 (Wang et al., 2018).

Accumulating evidence suggested that autophagy participated in the pathogenesis of PD. Autophagy dysregulation can impair many subcellular functions, including α-syn degradation (Lu et al., 2020). Based on the findings of Zhao et al., UNC51-like kinase (ULK1), a serine/threonine kinase responsible for promoting cell autophagy, was positively regulated by miRNA-132-5p (miR-132-5p; Zhao et al., 2020a). In addition, both miRNA-181b and miRNA-199a were able to mediate autophagy by targeting the PTEN/Akt/mTOR pathway. The downregulation of miRNA-181b in PD inhibited the Akt/mTOR signaling pathway, thereby improving autophagy (Li et al., 2018). Similarly, the downregulation of miRNA-199a in PD enhanced autophagy by regulating the activity of mTOR (Ba et al., 2020). Mitophagy is a form of autophagy in which cells remove damaged mitochondria to maintain cellular homeostasis. It has been reported that the accumulation of dysfunctional mitochondria would lead to the death of DA neurons (John et al., 2020). According to the research conducted by Zhou et al., miRNA-103a-3p (miR-103a-3p) was able to regulate mitophagy by mediating Parkin at the post-transcriptional level. Since Parkin is an E3 ubiquitin ligase that function in promoting the removal of damaged mitochondria *via* mitophagy, the upregulated level of miR-103a-3p in PD may harm the nervous system (Zhou J. et al., 2020).

In addition to autophagy dysregulation, dysregulated apoptosis had a crucial role in the pathogenesis of PD (Li D. et al., 2020). Although apoptosis was necessary for building neural networks, excessive apoptosis would accelerate the progression of PD (Liu et al., 2019). Three miRNAs were found to downregulate neuroprotective factors, including miRNA-183 (miR-183), miRNA-384-5p (miR-384-5p), and miRNA-421 (miR-421; Gao et al., 2019; Tao et al., 2020; Dong et al., 2021). Conversely, several miRNAs were able to inhibit neuronal apoptosis and therefore played a protective role in PD. Increasing evidence has shown that SNCA, Bcl-2-like protein 11 (BCL2L11), Ras-related C3 botulinum toxin substrate 1 (RAC1), Bax, caspase-3, and p53 were apoptotic activators. Their overexpression can be attenuated by microRNA-30b (miR-30b), microRNA-181c (miR-181c), microRNA-133a (miR-133a), microRNA-216a (miR-216a), let-7d, and microRNA-132 (miR-132), respectively (Li et al., 2017; Wei M. et al., 2017; Lu W. et al., 2020; Shen et al., 2020; Yang et al., 2020; Qazi et al., 2021). However, this effect may be diminished due to the downregulation of these six miRNAs in PD. p38 MAPK pathway has been proven to be important in regulating cell apoptosis. microRNA-599 (miR-599) and microRNA-181a (miR-181a) functioned in inactivating the p38 MAPK pathway, thereby protecting neurons (Liu Y. et al., 2017; Wu Q. et al., 2019). In addition, the activation of ERK1/2 and AMPK/mTOR pathways were essential to cell proliferation, which could be suppressed by microRNA-133b (miR-133b) and microRNA-185 (miR-185; Wen et al., 2018; Dong et al., 2020). miR-7 and microRNA-128 (miR-128) regulated the Wnt/beta-catenin signaling pathway differently. miR-7 suppressed the proliferation of DAs by inhibiting Wnt/beta-catenin pathway, whereas microRNA-128 (miR-128) alleviated the inhibiting effect of axis inhibition protein 1 (AXIN1) on this pathway and blocked DA apoptosis (Zhou et al., 2018; Adusumilli et al., 2020). microRNA-221 (miR-221) and miR-124 could extend cell lifespan *via* indirect regulation of PI3K/Akt and Hedgehog pathways (Li L. et al., 2018; Wang J. et al., 2019).

In recent years, the neuropathological consequences of dysregulated glutamate homeostasis have been recognized, and glutamate excitotoxicity has been associated with PD. Glutamate is an essential neurotransmitter in the mammalian central nervous system, responsible for transmitting excitatory signals between neurons. The amount of glutamate at the synapse above the physiological range was toxic and could have a detrimental effect on neuron cells, which was termed glutamate excitotoxicity (Iovino et al., 2020). Glutamate transporter-1 (GLT-1) was responsible for clearing excess glutamate from the synaptic gap to maintain glutamate homeostasis. It was found that GLT-1 mRNA was directly targeted by miRNA-543-3p (miR-543-3p). The overexpression of miR-543-3p in PD was able to suppress the expression and function of GLT-1 protein (Wu X. et al., 2019). miRNA-30a-5p (miR-30a-5p) was also upregulated in a mouse model of PD, while the regulatory mechanism of PKCα by miR-30a-5p needs further research. Meng et al. revealed that once PKCα was activated, it could induce ubiquitination and subsequent degradation of GLT-1 (Meng X. et al., 2021). Hence, both miR-543-3p and miR-30a-5p contributed to the pathology of PD by reducing the level of GLT-1, making them promising targets for treatment.

Briefly, numerous studies have validated those miRNAs were capable of regulating recognized causative factors of PD such as neuronal cell damage, microglia activation, and α-syn production, and chemical modifications by repressing the expression of target genes. The lncRNA can also influence the progress of this disease, however, it does not do so in the same way as the miRNA.

### Effects of lncRNAs in Parkinson’s disease

The progression of PD is normally accompanied by apoptosis and inflammation of nerve cells (Bhattacharyya et al., 2021). Two lncRNAs, namely nuclear-enriched abundant transcript 1 (NEAT1) and SNHG gene 14 (SNHG14), were identified to accelerate this progression (Zhou S. et al., 2020; Liu et al., 2021). The main hallmark of PD is the loss of dopaminergic neurons (DAs) in the substantia nigra, which correlates with typical PD symptoms including resting tremors and bradykinesia (Xin and Liu, 2021). SNHG14 reduced the number of DAsby sponging miR-133b, whereas H19 exerted a protective role against DAneuron damage (Zhang L. M. et al., 2019; Jiang J. et al., 2020). This protection was based on the overexpression of hypoxanthine-guanine phosphoribosyltransferase (HPRT), which was prompted by H19 sponging microRNA-301b-3p (miR-301b-3p; Jiang J. et al., 2020).

In PD, the main form of DA neuron death is apoptosis. Abnormalities in apoptosis are a sign of the loss of DAs in the substantia nigra, which have a notable effect on the development of PD. Zhang et al. found that microRNA-583-3p (miR-583-3p) downregulated the expression of PIK3R3. lncRNA H19 could attenuate the apoptosis of neurons by interacting with miR-583-3p (Zhang Y. et al., 2020). lncRNA myocardial infarction-associated transcript (MIAT) also exerted a neuroprotective role in PD. Shen et al. revealed that MIAT regulated synaptotagmin-1 (SYT1) by binding to microRNA-34-5p (miR-34-5p), which enhanced cell viability and inhibited apoptosis (Shen et al., 2021a). In contrast, growth arrest-specific 5 (GAS5) may cause loss of neuronal cells. The underlying mechanism was that GAS5 negatively regulated microRNA-150 (miR-150) and positively regulated fos-like antigen-1 (Fosl1), which resulted in cell apoptosis (Ma et al., 2022). In addition, a number of evidence indicated that LINC00943, long intergenic noncoding RNA-p21 (lincRNA-p21), NEAT1, and small molecule RNA host gene 1 (SNHG1) acted as molecular sponges of microRNA-338-3p (miR-338-3p), microRNA-1277-5p (miR-1277-5p), miR-124, microRNA-1301-3p (miR-1301-3p), and microRNA-216-3p (miR-216-3p), thereby inhibiting cell viability (Xu et al., 2018, Liu J. et al., 2020; Meng C. et al., 2021; Wang et al., 2021b). LRRK2 gene has been acknowledged to be associated with PD. Researchers found that Hox transcript antisense intergenic RNA (HOTAIR), X-inactive specific transcript (XIST), and metastasis-associated lung adenocarcinoma transcript 1 (MALAT1) indirectly enhanced the expression of LRRK2, which would increase the rate of apoptosis (Wang et al., 2017; Chen Q. et al., 2018; Zhou Q. et al., 2021). HOTAIR may also promote apoptosis of neuronal cells through microRNA-126-5p (miR-126-5p)/RAB3A-interacting protein (RAB3IP) axis (Lin et al., 2019). Furthermore, MALAT1 contributed to the apoptosis of DAs by combining with miR-124 and microRNA-135-5p (miR-135-5p; Liu W. et al., 2017; Lv et al., 2021).

Neuroinflammation is believed to play important role in the development of PD. Multiple proinflammatory cytokines released by activated microglia are involved in neuroinflammatory responses, which would eventually induce apoptosis of DA neurons (Xin and Liu, 2021). Cao et al. revealed that lncRNA HOXA11-AS activated microglia and promoted neuroinflammation *via* regulating miR-124-3p/NF-κB axis (Cao et al., 2021). Besides, lncRNA nuclear enriched abundant transcript 1 (NEAT1) acted as sponges of miR-124, microRNA-212-5p (miR-212-5p), and microRNA-519a-3p, thereby increasing the expression of phosphodiesterase 4B (PDE4B), RAB3A-interacting protein (RAB3IP), and specific protein 1 (SP1; Xie S. P. et al., 2019; Liu R. et al., 2020; Chen M. Y. et al., 2021). Since PDE4B, RAB3IP, and SP1were shown to facilitate cell inflammation, the knockdown of NEAT1 could be a potential strategy for treating PD patients. Based on the study of Sun et al., LINC00943 also positively regulated SP1 expression, making it a possible therapeutic target in PD (Sun et al., 2022). Small molecule RNA host gene 1 (SNHG1) was found to prevent microRNA-181a-5p (miR-181a-5p) from suppressing C-X-C motif chemokine 12 (CXCL12). This may trigger inflammatory responses (Wang et al., 2021a). In addition, SNHG1 as well as growth arrest-specific 5 (GAS5) had a promotion effect on the expression of an inflammasome named nod-like receptor protein 3 (NLRP3), which would stimulate the secretion of inflammatory factors (Cao et al., 2018; Xu et al., 2020).

Abnormal aggregation of α-syn also causes damage to DA neurons. Liu et al. discovered that the expression of nuclear-enriched abundant transcript (NEAT) was positively correlated with the α-syn expression, suggesting that the knockdown of NEAT may protect neurons (Liu and Lu, 2018). Both UCA1 and small nucleolar RNA host gene 1 (SNHG1) were able to promote the accumulation of α-syn. Based on the study of Lu et al., UCA1 could upregulate the expression of SCNA (Lu et al., 2018). In addition, Chen et al. found that the inhibitory effect of microRNA-15b-5p (miR-15b-5p) on the expression of seven in absentia homolog 1 (SIAH1) could be reversed by SNHG1. The overexpression of SIAH1 would induce the aggregation of α-syn and elevate its toxicity (Chen Y. et al., 2018).

Autophagy is responsible for the degradation of α-syn. The dysregulation of this process is an important contributor to the development of PD (Xin and Liu, 2021). Yan et al. revealed that lncRNA nuclear paraspeckle assembly transcript 1 (NEAT1) might promote autophagy in PD by stabilizing PTEN-induced kinase 1 (PINK1; Yan et al., 2018). Brain-derived neurotrophic factor anti-sense (BDNF-AS) was also able to promote autophagy. According to the study by Fan et al., BDNF-AS enhanced the number of autophagosomes by regulating microRNA-125b-5p (miR-125b-5p) negatively (Fan Y. et al., 2020). In contrast, SNHG1 was found to increase the expression level of p27 *via* sponging microRNA-221/222 (miR-221/222). Since p27 was believed to have a role in inhibiting autophagy, the downregulation of SNHG1 may promote autophagic activation (Qian et al., 2019). PLK2 was associated with a pathway that inducedα-syn degradation *via* autophagy. microRNA-126 (miR-126) suppressed the expression of p27, which could be blocked by Opa interacting protein five antisense RNA 1 (OIP5-AS1). Hence, OIP5-AS1 exerted a protective role in PD by accelerating the clearance of α-syn (Song and Xie, 2021). Based on the findings of Zhao et al., OIP5-AS1 also had a role in promoting mitochondrial autophagy, a process that selectively removes unwanted or damaged mitochondria. Specifically, the expression of NIX was down-regulated by microRNA-137 (miR-137), which was reversed by OIP5-AS1. Overexpression of NIX was proved to promote mitochondrial autophagy, which would prevent neuronal death (Zhao et al., 2022). For further review on targeting α-syn as a therapy for PD, please refer to Taylor et al. (2002), Wong and Cuervo (2010), Vidal et al. (2014), Martire et al. (2015), Pickrell and Youle (2015), Dunn et al. (2019), and Fields et al. (2019).

To sum up, lncRNAs have been shown to affect apoptosis and autophagy of neurons, the accumulation and degradation of α-syn, and neuronal inflammation. The regulatory roles they play in PD are largely accomplished by sponging miRNAs.

The *in vitro* and *in vivo* experiments have indicated the feasibility of treating PD by targeting miRNAs and lncRNAs. These regulatory ncRNAs may serve as targets in PD treatment, while the effectiveness and safety of this therapy have yet to be tested in human trials. Other regulatory ncRNAs, such as circRNAs, may sponge specific miRNAs, thus contributing to the development of PD (Lu et al., 2019; Feng et al., 2020; Wang W. et al., 2021). Further research is needed to unravel the role of a wider class of regulatory ncRNAs in PD progression, which would not only further our understanding but also lead to the development of novel and effective therapeutic strategies (Acharya et al., 2020).

## Toxicological Effects of Oxidative Stress in Parkinson’s Disease

**Figure 1:**
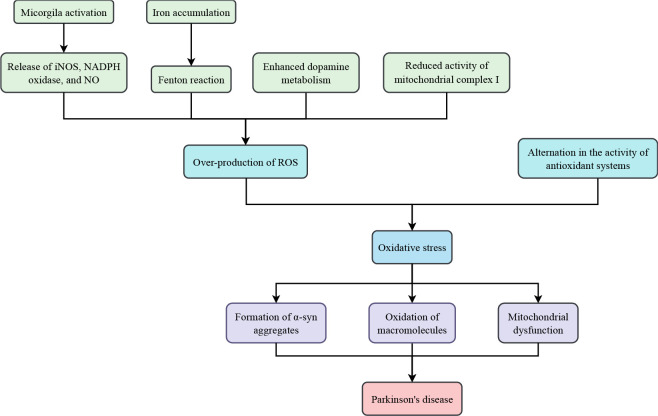
Oxidative stress in Parkinson’s disease.

ROS generated in the body perform physiologic functions such as stimulating growth factors, promoting inflammatory responses, and regulating cell production. However, when their levels far exceed that of antioxidants, human cells will be subjected to devastating effects (Surendran and Rajasankar, 2010; Zuo and Motherwell, 2013; Hemmati-Dinarvand et al., 2019). Neurons are likely to be attacked by oxidative stress since they consume large amounts of oxygen and possess relatively modest levels of antioxidant enzymes. Accumulating evidence has demonstrated that oxidative stress is an important factor in the etiology and progression of PD (Figure 1; Percario et al., 2020).

### Oxidative stress

**Figure 2:**
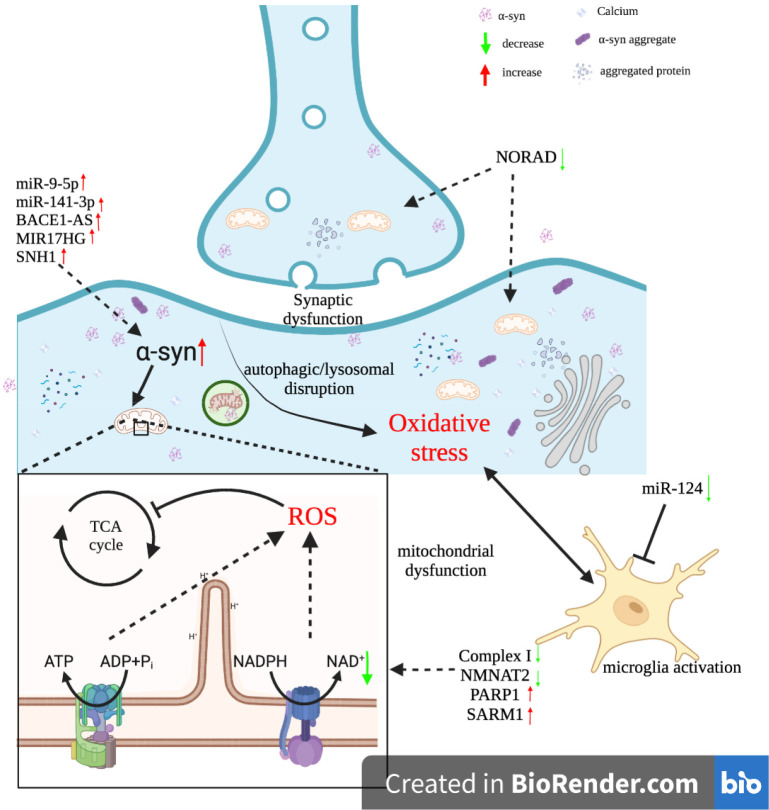
Interactions between ncRNAs and oxidative stress in Parkinson’s disease.

Mitochondrial dysfunction was found in the substantia nigra (SN) in some patients with PD. Although the mechanism by which mitochondrial depletion causes oxidative stress and bioenergetic deficiency is not fully understood, researchers have correlated the impairment or inhibition of mitochondrial complex I with elevated levels of ROS (Schapira et al., 1990a, b, c). Complex I (NADH-ubiquinone oxidoreductase) is a major component of the oxidative phosphorylation system responsible for converting molecular oxygen into water and driving energy synthesis (Hauser and Hastings, 2013). Reduced activity of complex I may lead to disruption of electron transfer, which would result in excessive ROS production (Figure 2; Sarkar et al., 2016).

Mitochondrial-related energy failure may also disrupt the vesicular storage of DA (Puspita et al., 2017). Under normal circumstances, dopamine was preserved in synaptic vesicles, which were an acidic and stable environment that protected dopamine from oxidation (Jin et al., 2014). In the SN of PD patients, however, a rise in the amount of free dopamine in the cytoplasm has been observed (Sackner-Bernstein, 2021). Monoamine oxidases (MAO) functioned in catalyzing the transition from cytosolic dopamine to H_2_O_2_and 3,4-dihydroxyphenylacetaldehyde (DOPAL; Raza et al., 2019; Zaman et al., 2021). In addition, dopamine may undergo auto-oxidation to form DA quinones (Janda et al., 2012; Smeyne and Smeyne, 2013). H_2_O_2_ was a by-product of both of these reactions, which could be further transformed into •OH *via* the Fenton reaction (Vallee et al., 2020). This reaction was largely dependent on the presence of iron, which was also identified to be elevated in the PD SN (Vallée et al., 2021a). Therefore, enhanced dopamine metabolism in combination with iron accumulation may contribute to cellular ROS in dopaminergic neurons.

In addition to the alternations occurring within neuronal cells, microglia activation has been regarded as an essential contributor to ROS production (Hassanzadeh and Rahimmi, 2018). Usually, microglia cells, the main immune cells in the brain, remained quiescent. Once activated, microglia released ROS and RNS such as H_2_O_2_, O^•–^_2_, and NO (Onyango, 2008). The reaction of the latter two free radicals produced peroxynitrite (ONOO^−^), a highly reactive molecule that may induce apoptosis of DA neurons (Varcin et al., 2012). Furthermore, the capacity of activated microglia to produce ROS and RNS was enhanced due to the increased release of inducible nitric oxide synthase (iNOS) and NADPH oxidase. These two enzymes promoted the generation of O^•–^_2_ and NO, respectively (Drechsel and Patel, 2008; Koppula et al., 2012). Excessive intracellular and intercellular free radicals would lead to oxidative stress (Hald and Lotharius, 2005).

To deal with high contents of ROS and protect themselves from oxidative damage, neuronal cells utilized an antioxidant system consisting of antioxidant enzymes in conjunction with low molecular compounds (Manoharan et al., 2016). The enzymes included catalase (CAT), superoxide dismutase (SOD), and glutathione peroxidase (GPx), whereas glutathione (GSH) was a major non-enzymatic antioxidant (Redensek et al., 2019; Robea et al., 2020). CAT and GPx were believed to be responsible for scavenging H_2_O_2_ (Foley and Riederer, 2000). In the substantia nigra pars compacta (SNc) of PD patients, however, a dramatic decrease in the activity of antioxidative enzymes and the levels of non-enzymatic antioxidants has been observed (Damier et al., 1993; Mythri et al., 2011). Such an imbalance may induce oxidative stress and accelerate PD progression (Hauser and Hastings, 2013).

### Oxidative stress accelerates the progression of Parkinson’s disease

Oxidative stress correlates with increased oxidation of macromolecules, including lipid, nucleic acid, and protein (Vallée et al., 2021a). Lipids are components of cell membranes, maintaining membrane fluidity and permeability. Hence, lipid oxidation would directly cause structural damage to cell membranes, which may lead to neuronal damage or even death (Dalle and Mabandla, 2018). Elevated oxidative damage to nucleic acids was also revealed in the PD SN (Hegde et al., 2006). The conformation and stability of DNA were altered because of oxidative stress, which could result in cell death (Guo et al., 2018). Protein oxidation is believed to be a feature of oxidative damage in the PD SN (Taylor et al., 2013). Oxidative stress caused nitration or carbonylation of proteins, which may lead to loss of function or aggregation (Maguire-Zeiss et al., 2005). The cellular systems responsible for the removal of misfolded or aggregated proteins were impaired by oxidative stress, which also contributed to the formation of protein aggregates (Hassanzadeh and Rahimmi, 2018).

Mitochondria are the main source of ROS in cells and are highly vulnerable to oxidative damage (Yuan et al., 2007). A dramatic outbreak of free radicals impaired the capability of the ETC to transfer electrons, which would result in a steady decline in mitochondrial activity and increased ROS production (Subramaniam and Chesselet, 2013). Significant elevation of ROS levels in neurons was found to be responsible for GSH leakage, mitochondrial DNA (mtDNA) mutation, and DA oxidation, which further promoted the production of free radicals (Janda et al., 2012; Yan et al., 2013; Vallée et al., 2021a, b). The damage to neurons caused by the positive feedback loop consisting of these PD elements will eventually lead to apoptosis (Trist et al., 2019).

Oxidative stress was also found to induce the formation of α-syn aggregates. Under normal physiological conditions, the α-syn existed as monomers or tetramers (Figure 2). Due to sensitivity to the excessive accumulation of ROS, the α-syn was induced to misfold by oxidative stress (Tsang and Chung, 2009). Misfolded α-syn proteins formed oligomers or fibrils and eventually insoluble aggregates (Jiang et al., 2016). The degradation of α-syn aggregates within Das is dependent mainly on the ubiquitin-proteasomal system (UPS) or chaperone-mediated autophagy (CMA). However, in a highly oxidized environment, these two pathways became ineffective in mediating the degradation of α-syn aggregates (Ganguly et al., 2017). This is because oxidative stress along with α-syn proteins subjected to oxidative modifications impaired the UPS and CMA (Jimenez-Moreno and Lane, 2020). α-syn accumulation and aggregation were found to inhibit the synthesis of ATP by mitochondria and induce microglia activation, which led to chronic effects of oxidative stress on the SN (Maguire-Zeiss et al., 2005). Hence, the interaction between oxidative stress and α-syn proteins can be regarded as a positive feedback loop that drives pathological conditions, which ultimately leads to the development of PD (Puspita et al., 2017).

**Figure 3:**
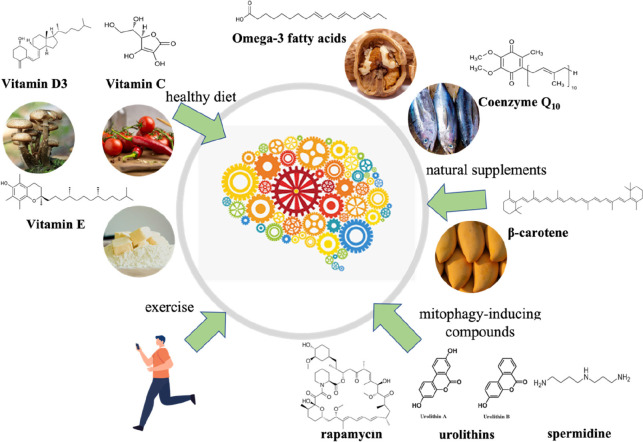
Multiple antioxidant strategies to prevent and cure Parkinson’s disease.

PD remains an incurable neurodegenerative disease, and the etiologies of it is not completely understood (Jiang et al., 2016; Raza et al., 2019). However, since researchers have found that oxidative stress can trigger PD or accelerate its progression, there is a consensus that combating oxidative stress is a promising medicinal strategy (Janda et al., 2012). Many molecules and natural compounds exert antioxidant properties, including carvacrol, coenzyme Q10 (CoQ10), creatine, curcumin, melatonin, lipoic acid (LA), lycopene, N-acetyl-cysteine (NAC), vitamin B_3_, vitamin C, vitamin D_3_, and urate (Chen et al., 2012; Crotty et al., 2017; Ciulla et al., 2019; Figure 3). These antioxidants may serve as neuroprotective agents, while they need to be proven safe and effective in clinical trials (Henchcliffe and Beal, 2008; Hassanzadeh and Rahimmi, 2018). In addition, regular physical exercise has been identified to have a positive impact on PD *via* reducing oxidative stress (Fan B. et al., 2020; Robea et al., 2020).

## Interaction Between Oxidative Stress and Regulatory Ncrnas in Parkinson’s Disease

As mentioned above, both regulatory non-coding RNAs, as well as oxidative stress, are closely associated with PD. Furthermore, oxidative stress can cause oxidative damage to RNA. In contrast, regulatory ncRNAs such as miRNAs and lncRNAs play a role in regulating ROS production (Figure 2). Their interactions have been confirmed to be involved in the pathophysiology of PD (Konovalova et al., 2019).

### RNA oxidation

RNA is susceptible to oxidative stress due to its single-stranded structure and dense distribution near the mitochondria, where most intracellular ROS are generated (Nunomura et al., 2009; Liu Z. et al., 2020). Excessive amounts of ROS may lead to RNA strand breaks and chemical modification and excision of RNA bases (Song et al., 2011; Zhao et al., 2017). Due to the lack of advanced repair mechanisms, oxidatively damaged RNA would accumulate in cells, resulting in reduced protein synthesis, erroneous protein generation, and eventual cell death (Zhang et al., 1999; Nunomura et al., 2006). RNA oxidation is not only a common feature of PD but also an early event in the progression of this disease (Nunomura et al., 2007; Cervinkova et al., 2017). Among oxidative marks on RNA, 8-oxo-7, 8-dihydroguanosine (8-oxoG) might be the most abundant and most extensively studied one (Gonzalez-Rivera et al., 2020). This base adduct can be produced by the exposure of guanine to free radicals and may cause incorrect base pairing (Zhang and Li, 2020). Researchers have found that 8-OHG levels in cerebrospinal fluid (CSF) and serum are significantly higher in PD patients than in healthy controls, indicating that 8-OHG may serve as a biomarker for PD (Alam et al., 1997; Kikuchi et al., 2002; Abe et al., 2003).

Non-coding RNAs, which are not responsible for encoding proteins, make up the majority of RNAs in human cells (Moreira et al., 2008). As a category of ncRNAs, regulatory ncRNAs, including miRNAs, lncRNAs, and circRNAs, are involved in the regulation of gene expression (Kong and Lin, 2010). Compared to mRNAs, these regulatory ncRNAs live relatively longer. Hence, oxidative damage that impairs their function would have a detrimental effect on cellular homeostasis (Yan and Zaher, 2019). For example, miRNAs attacked by free radicals may fail to correctly recognize their target mRNAs, which may lead to increased expression of certain proteins (Nunomura and Perry, 2020). In the experiment conducted by Je and Kim, miR-7 and miR-153 were identified to suppress the expression of α-SYN. Their mediated translational inhibition was abolished by oxidative stress, resulting in increased α-SYN levels and subsequent development of PD (Je and Kim, 2017). Furthermore, the study of Chen et al. showed that oxidative stress induced N6-methyladenosine (m^6^A) modification of circRNAs. m^6^Amodified circRNAs influenced the expression of stress response genes, which could be a potential mechanism for oxidative stress-induced neurodegenerative diseases (Chen N. et al., 2021).

### miRNAs regulate oxidative stress

α-syn is responsible for inducing oxidative stress. Both microRNA-141-3p (miR-141-3p) and microRNA-9-5p (miR-9-5p) were found to target the 3’ UTR of SIRT1 mRNA. Since SIRT1 inhibited the formation of α-syn aggregates, knockdown of miR-141-3p and miR-9-5p may alleviate oxidative stress and boost the viability of Das (Wang Z. et al., 2019; Zheng et al., 2020). In addition, microglia are thought to have a role in the pathophysiology of PD, since cytotoxic substances released from activated microglia can exacerbate oxidative stress. miR-124 inhibited microglia activation, thereby representing a neuroprotective factor (Lushchak and Lushchak, 2021). Recently, researchers found that dysregulation of Fe^2+^ homeostasis may lead to the accumulation of ROS in cells. This homeostasis was maintained by ferritin heavy chain 1 (FTH1) since FTH1 converted Fe^2+^ ions into soluble, non-toxic Fe^3+^ ions. microRNA-335 (miR-335) suppressed the expression of FTH1, thereby promoting the release of Fe^2+^ ions and the generation of free radicals (Li et al., 2021). Furthermore, downregulation of microRNA-410 (miR-410) expression in PD was identified to be associated with elevated ROS production, although the underlying mechanism by which miR-410 exerted its neuroprotective role needs further study (Ge et al., 2019).

SOD, CAT, and GPx are responsible for detoxifying oxidants and repairing oxidative damage. Based on some research, microRNA-375 (miR-375), microRNA-218-5p (miR-218-5p), and miR-185 attenuated oxidative stress, as evidenced by elevated SOD and GPx activity in PD rats treated with these miRNAs (Cai et al., 2020; Ma et al., 2021b; Qin et al., 2021). In contrast, miR-137 and microRNA-494-3p (miR-494-3p) aggravated oxidative stress by reducing the level of SOD (Geng et al., 2018; Jiang et al., 2019). The activity of SOD was down-regulated by microRNA-155-5p (miR-155-5p; Lv et al., 2020).

### lncRNAs regulate oxidative stress

Mitochondrial dysfunction, a common feature of PD, is directly related to the excessive production of ROS. This process can be inhibited by upregulated NORAD (Song et al., 2019). In addition, the formation of α-syn aggregates is capable of exacerbating oxidative stress *via* downregulating complex I activity or activating microglia. Li et al. found that the upregulation of lncRNA beta-amyloid cleaving enzyme-antisense (BACE1-AS) in PD wasassociatedwithrisingα-syn levels (Li Y. et al., 2020). Besides, Zhang et al. discovered that the lncRNA miR-17-92a-1 cluster host gene (MIR17HG) played a role in promoting the expression of α-syn. MIR17HG sponged microRNA-153-5p (miR-153-5p), thereby preventing miR-153-5p from downregulating α-syn expression (Zhang et al., 2022). GSK3β was revealed to promote α-syn accumulation *via* inhibiting autophagy. The expression of GSK3β was suppressed by miR-15b-5p, which was reversed by SNHG1 binding to miR-15b-5p (Xie N. et al., 2019).

Among the 17 newly studied lncRNAs, 13 were identified to aggravate oxidative stress and inflammatory responses in neurons, namely AL049437, HOTAIR, LINC00943, lncRNA-p21 (lnc-p21), MIAT, NEAT1, rhabdomyosarcoma 2-associate transcript (RMST), SNHG1, SNHG7, SOS1 intronic transcript 1 (SOS1-IT1), SRY-box transcription factor 2 overlapping transcript (SOX2-OT), taurine upregulated gene 1 (TUG1), and UCA1 (Cai et al., 2019; Ding et al., 2019; Zhai et al., 2020; Zhang L. et al., 2020; Zhao et al., 2020b; Guo et al., 2021; Lian et al., 2021; Ma et al., 2021a; Zhang et al., 2021; Zhou S. et al., 2021; Fan et al., 2022; Lang et al., 2022). The other four lncRNAs, namely JHDM1D antisense 1 (JHDM1D-AS1), myocardial infarction associated transcript 2 (Mirt2), small nucleolar RNA host gene 12 (SNHG12), and PART1, exhibited anti-oxidant and anti-inflammatory roles in models of PD, as evidenced by the decline in proinflammatory cytokines and increase in SOD contents (Han et al., 2019; Shen et al., 2021b; Wang C. et al., 2021; Yan et al., 2021). Nevertheless, the precise mechanism by which these 17 RNAs affect oxidative stress and thereby regulate PD progression remains unclear.

At present, we are in the early stage of investigating the cellular consequences of oxidatively damaged RNA and the mechanism by which regulatory ncRNAs affect oxidative stress (Kong et al., 2008; Xu et al., 2021). Further investigations are needed to explore the association between regulatory ncRNA oxidation and PD, which is beneficial to the development of early diagnosis and treatment for this disease (Nunomura et al., 2017).

## Conclusion

We described the role of regulatory ncRNAs, oxidative stress, and their interactions in the regulation of PD. In recent years, the intrinsic correlation between regulatory ncRNAs and oxidative stress in PD has been increasingly studied. It is worth pointing out that some regulatory ncRNAs have been found to influence the progression of PD *via* regulating oxidative stress, which makes them potential therapeutic targets. However, there is fairly limited research that uncovers the precise mechanisms. In addition, because of the differences between the brains of laboratory animals and the human brain in combination with the inability of experimental models to accurately recapitulate the various features of PD, translating the results from PD models to humans may face considerable difficulties (Chia et al., 2020). There are still many hurdles to be overcome in the study of the interplay between regulatory ncRNAs and oxidative stress in PD. With the continuous innovation of experimental methods and techniques, the application of safe and effective targeted drugs for PD treatment is foreseeable.

## Author Contributions

The outline of this review was conceived by MT and XG. The draft manuscript was accomplished by HZ and polished by XL and GL. The tables were provided by JL and the figures were constructed by YL. All authors made a direct and intellectual contribution to this topic. All authors contributed to the article and approved the submitted version.

## Funding

This work was financially supported by grants from the National Natural Science Foundation of China (32002235).
